# Qualitative and quantitative analysis of aerosol and droplet dispersion during simulated dental implant procedures using three types of instruments

**DOI:** 10.1007/s00784-025-06583-z

**Published:** 2025-10-06

**Authors:** Hiromitsu Morishima, Tomonari Kajita, Jun Watanabe, Kenji Kikuchi, Yoko Iwamatsu-Kobayashi, Wataru Yashiro, Hiroyasu Kanetaka, Hiroshi Egusa, Kensuke Yamauchi

**Affiliations:** 1 Division of Oral and Maxillofacial Reconstructive Surgery, Graduate School of Dentistry, Tohoku University, 4-1 Seiryo-machi, Aoba-ku, Sendai, 980-8575 Miyagi Japan; 2 Division of Oral and Maxillofacial Oncology and Surgical Sciences, Graduate School of Dentistry, Tohoku University, Sendai, Miyagi Japan; 3 Division of Dental Safety and System Management, Tohoku University Hospital, Sendai, Miyagi Japan; 4 Division of Molecular and Regenerative Prosthodontics, Graduate School of Dentistry, Tohoku University, Sendai, Miyagi Japan; 5 Biological Flow Studies Laboratory, Department of Finemechanics, Graduate School of Engineering, Tohoku University, Sendai, Miyagi Japan; 6 Next-Generation Detection System Smart Lab, International Center for Synchrotron Radiation Innovation Smart (SRIS), Tohoku University, Sendai, Miyagi Japan; 7 Frontier Quantum-beam Metrology Laboratory, Institute of Multidisciplinary Research for Advanced Materials (IMRAM), Tohoku University, Sendai, Miyagi Japan; 8 Department of Applied Physics, School of Engineering, The University of Tokyo, Tokyo, Japan; 9 Liaison Center for Innovative Dentistry, Graduate School of Dentistry, Tohoku University, Sendai, Miyagi Japan; 10 Division of Orthodontics and Dentofacial Orthopedics, Graduate School of Dentistry, Tohoku University, Sendai, Japan

**Keywords:** Aerosol, Droplets, Dental implant surgery, Simulated dental treatment, High-speed digital camera, Extraoral vacuum

## Abstract

**Objectives:**

This study aimed to investigate the generation and dispersion dynamics of aerosols and droplets produced during dental procedures, including implant surgery.

**Materials & methods:**

Dental procedures were simulated on a test model using three different instruments: an air turbine handpiece, an ultrasonic device, and an implant motor. Particle behavior was visualized using two types of illumination light sources combined with a high-speed digital camera, enabling both qualitative and quantitative assessments of aerosol and droplet dispersion. Additionally, droplet deposition on water-sensitive paper placed in three different locations was analyzed to compare dispersion patterns among the three instruments.

**Results:**

The air turbine handpiece produced the highest luminance intensity (mean ± SD: 112.3 ± 6.4 a.u., *n* = 9), which was significantly greater than that of the implant motor (78.5 ± 5.2 a.u., *n* = 9; *p* < 0.05). For all devices, droplet diffusion was lower during molar treatment than during anterior tooth procedures. Water-sensitive paper analysis revealed increased droplet deposition at the extraoral vacuum site when the vacuum was activated (air turbine: 62 droplets; ultrasonic device: 49 droplets; *n* = 3 trials each), whereas droplet counts decreased at the patient’s forehead.

**Conclusions:**

Simulated implant surgery generated less droplet dispersion compared with other dental procedures. Furthermore, the use of an extraoral vacuum markedly reduced droplet spread during various dental treatments.

**Clinical relevance:**

These data support layered controls—judicious instrument selection and extraoral suction—to reduce exposure during aerosol-generating procedures. Findings derive from a standardized simulation and should be validated in clinical settings.

## Introduction

Aerosols and droplets produced during dental care have been identified as potential carriers for infectious agents between individuals [1]. SARS-CoV-2, the virus responsible for COVID-19, resides in the upper respiratory tract and can also be detected in saliva, often at levels similar to those in nasopharyngeal secretions [2]. Virus-laden aerosols and droplets therefore play a critical role in transmission. Dental procedures frequently employ air turbine handpieces and ultrasonic devices, which are known to generate considerable aerosols containing saliva, blood, or dental plaque, and thus heighten the potential for airborne cross-infection [3, 4]. Better understanding of aerosol transmission mechanisms and their clinical implications is essential to minimize bacterial and viral infections arising from droplets during routine dental care.

It has been reported that during third molar extraction with an air turbine, aerosols containing blood may disperse to distances of up to one meter from the surgical site [5]. Studies, both bacteriological and observational, indicate that assistants as well as operators can be exposed to aerosols generated during dental treatment [6]. Another study indicated that contamination is most likely to occur within 50 cm of the surgical field, though it can extend beyond 1 m [7]. These findings have heightened interest in elucidating how droplets containing pathogens such as SARS-CoV-2 and other microorganisms spread in clinical settings.

Nishimura et al. analyzed the dynamics of aerosol particles generated during sneezing and coughing using high-speed video imaging [8]. Accordingly, visualization of aerosol and droplet dynamics during dental treatment has become an important tool for understanding clinical risks. Implant surgery, in particular, carries exposure risk to contaminated aerosols containing blood and saliva, since it requires the use of devices such as implant motors and ultrasonic instruments. Although some studies have reported that reducing motor speed can decrease aerosol generation by implant devices [9], and others have examined aerosol dispersion during dental therapy [10], few investigations have directly compared the risks of aerosol and droplet generation across different dental procedures, especially between general dentistry and implant surgery.

A systematic review by Innes et al. found that instruments such as air turbine handpieces and ultrasonic scalers generate significantly more aerosols compared with other devices, highlighting variability in aerosol production depending on the instrument [11]. However, many aspects of this variability remain unclear, and detailed comparisons under standardized conditions are scarce. While high-speed imaging has advanced the visualization of aerosol behavior, direct comparative data across instruments remain limited. Furthermore, although extraoral vacuum systems are widely used, their clinical effectiveness and practicality require further evaluation.

Therefore, this study aimed to (1) compare aerosol and droplet dispersion generated by three dental instruments—an air turbine handpiece, an ultrasonic cutting device, and an implant motor—and (2) assess the effect of extraoral vacuum use in reducing dispersion. These instruments serve distinct clinical purposes, yet their aerosol-generating characteristics provide important insights into occupational risks.

The novelty of this study lies in its dual qualitative and quantitative assessment using high-speed imaging and droplet-sensitive media. The findings contribute new knowledge regarding exposure risks and potential mitigation strategies in a standardized dental simulation environment.

## Materials and methods

### Experimental setting and instruments

**Fig. 1:**
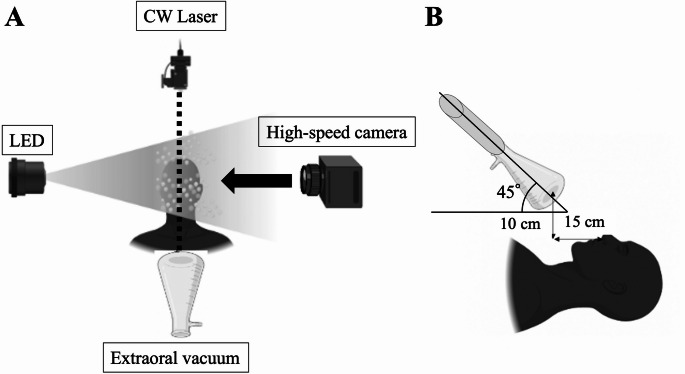
Positioning of devices for droplet visualization, (**A**) setup using LED illumination for qualitative assessment and a continuous-wave laser with a high-speed camera for quantitative assessment, arrow indicates the imaging direction, (**B**) lateral view showing the extraoral vacuum nozzle positioned at a 45° angle, 10 cm above and 15 cm in front of the patient’s mouth

Experiments were conducted in the laboratory of the Graduate School of Dentistry, Tohoku University. An overview of the experimental setup is presented in Fig. 1A and B. A mannequin simulating the upper body of a patient was placed in a supine position on a dental chair (EOM-PLUS SC, GC Corporation, Japan). Dental procedures were performed using three instruments: an air turbine handpiece (Green Impulse X-ML, GC Corporation, Japan; maximum speed 300,000 rpm), an ultrasonic (US) cutting device (VarioSurg 3, NSK, Japan; frequency 28–32 kHz), and an implant motor (Surgic Pro, NSK, Japan; maximum speed 1200 rpm). Procedures were performed on the buccal side of the mannequin’s oral cavity with continuous water injection.

Suction devices included a surgical intraoral suction tube integrated into the dental chair and a mobile extraoral vacuum (Free arm ARTEO-S, Tokyo Giken Co., Ltd., Japan; flow rate 3.0 m³/min). Intraoral suction was consistently used in all experiments, whereas the extraoral vacuum was tested under two conditions: activated (on) and deactivated (off). Qualitative and quantitative evaluations of aerosol and droplet dispersion were performed using high-speed digital imaging combined with two types of illumination.

### Qualitative assessments of aerosols and droplets

A light-emitting diode (LED) illumination system was employed to visualize diffusion of aerosol particles < 10 μm and droplets ranging from 20 μm to several millimeters generated by each instrument. The primary outcome was the visible direction and spread pattern of droplets emitted from the instrument tip. High-resolution images were obtained with a high-speed video camera system (Fastcam Mini AX, Photron, Japan) equipped with a 528-nm LED light source (IL-106G Illuminator, HARDsoft Microprocessor Systems, Krakow, Poland), recording at 1000 frames/s.

### Quantitative assessments of aerosols and droplets

Quantitative evaluation of aerosol spread was performed using a continuous-wave (CW) laser (Ventus 532, Laser Quantum; wavelength 532 nm, output 4.3 W) in combination with the same high-speed video system. Image processing steps included digital enhancement, background subtraction, and luminance thresholding with FIJI/ImageJ software (v1.53c, NIH, Bethesda, MD, USA) to isolate and measure aerosol spread.

**Fig. 2:**
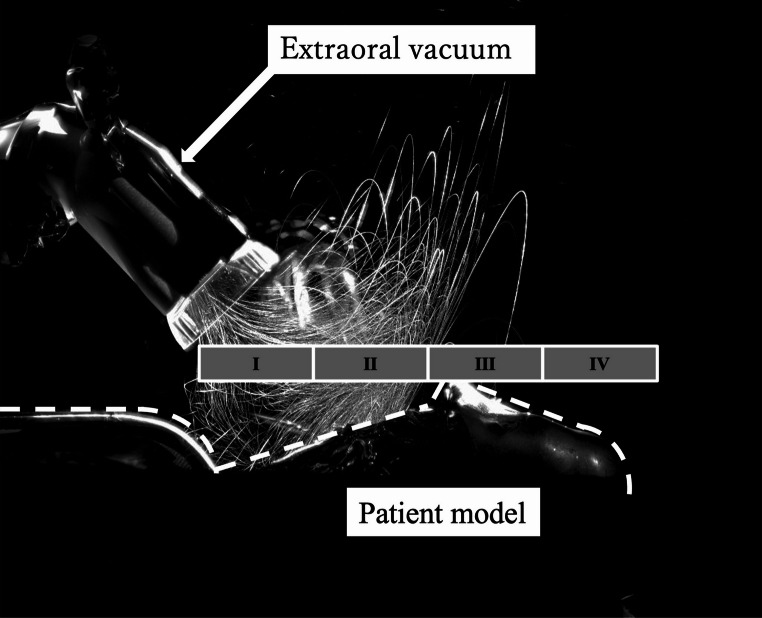
Schematic diagram of analysis areas, I submentum, II mouth, III nose, IV glabella

The dispersal area was defined as the maximum luminance intensity region and expressed in arbitrary units (a.u.). For spatial analysis, the patient’s craniofacial region was divided into four anatomical zones (Fig. 2).

- Area I: Submentum.
- Area II: Mouth.
- Area III: Nose.
- Area IV: Glabella.

When the operator was positioned at 12 o’clock relative to the patient’s cranio-caudal axis, the risk of exposure was assumed to increase progressively from Area I to Area IV.

### Quantitative assessments using water-sensitive paper

Droplet spread was further assessed using water-sensitive paper (WSP; 26 × 76 mm, Quantifoil Micro Tools GmbH, Germany), which stains blue upon contact with droplets > 50 μm in diameter. After exposure, ImageJ was used to measure droplet diameters (µm) and count the number of droplets from the WSP images. These data were then used for statistical analysis.

Papers were placed at three locations: (1) the extraoral vacuum site, (2) the patient’s forehead, and (3) the operator’s mask. Measurements were performed under two conditions (with and without extraoral vacuum activation).

**Fig. 3:**
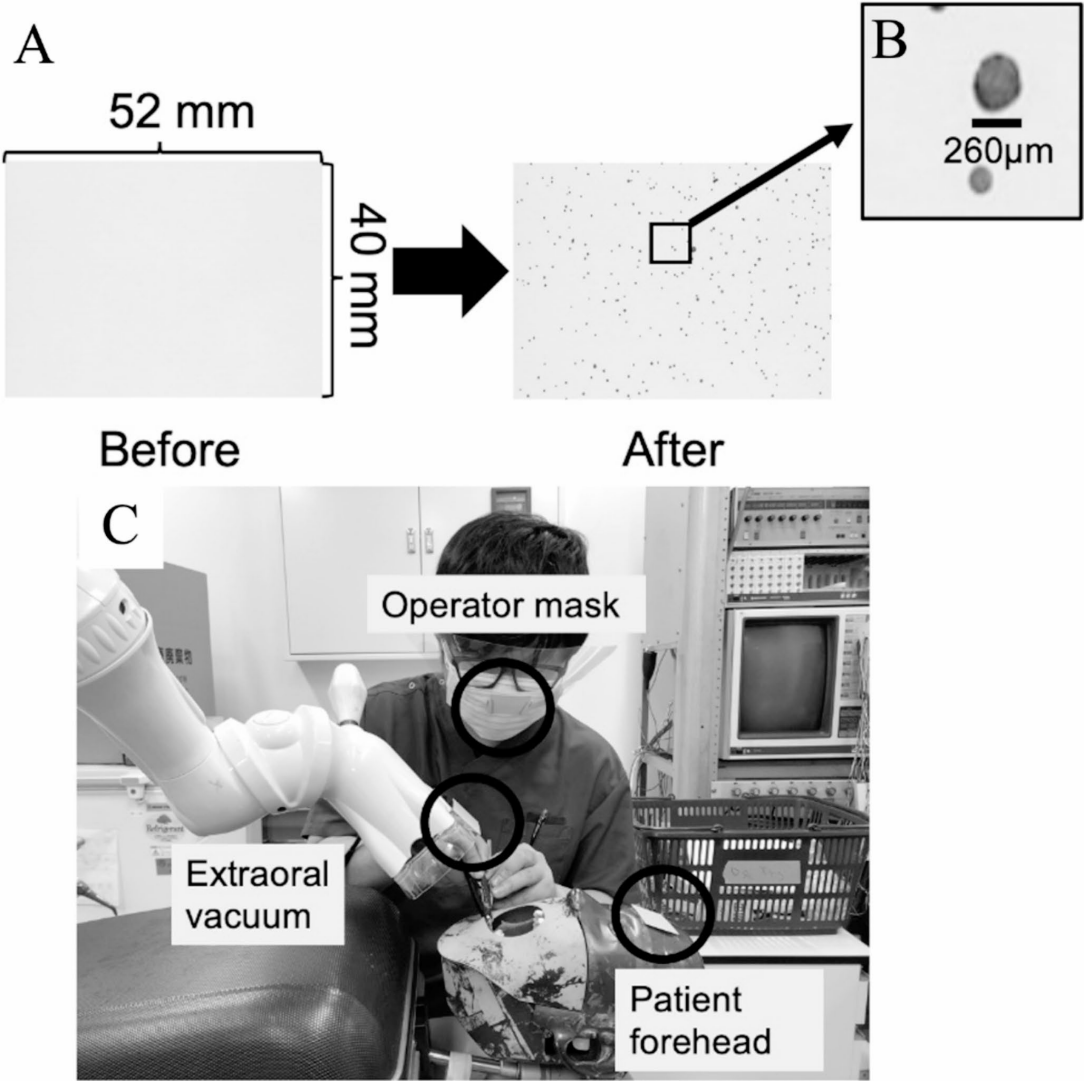
Water-sensitive paper method and experimental placement, (**A**) representative sheets before and after droplet exposure, (**B**) close-up showing individual droplet imprints with scale bar, (**C**) placement around the operator and patient for spatial analysis of droplet dispersion

During each procedure, water-sensitive paper was positioned for 30 s. For each condition, three trials were conducted, and the total droplet counts at each site were analyzed. Representative images included (A) unstained paper, (B) stained paper (close-up), and (C) paper placement (Fig. 3).

### Statistical analysis

Data were analyzed using one-way ANOVA followed by Tukey’s post hoc test for multiple comparisons among instruments. Tukey’s method was chosen over Bonferroni to preserve statistical power. To assess the effect of extraoral vacuum use and differences between molar and anterior tooth procedures, Student’s t-tests were performed.

Comparisons among instruments were conducted separately for molar and incisor sites; however, interaction effects could not be modeled, which is acknowledged as a limitation. All continuous variables were expressed as mean ± standard deviation (SD). A p-value < 0.05 was considered to indicate statistical significance.

## Results

### Visualization of droplet dispersion during dental treatment

**Fig. 4:**
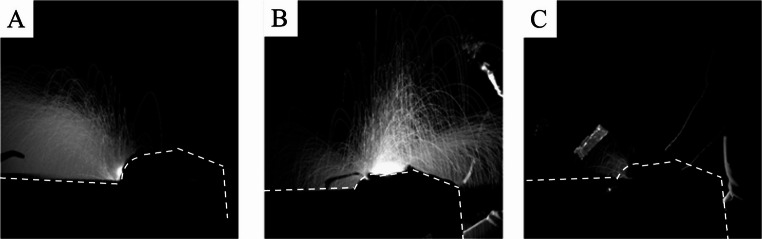
LED-illuminated photographs of aerosol and droplet dispersion without surgical intraoral suction or extraoral vacuum, (**A**) air turbine handpiece, (**B**) ultrasonic bone-cutting device, (**C**) implant motor

For this analysis, procedures were performed on the mandibular anterior teeth without activation of the extraoral vacuum. An LED light illuminated the operative field, and dispersed droplets were visualized by reflected light captured with a high-speed camera. Each instrument was assessed over a 20-second interval. Representative images are shown in Fig. 4.

Clear differences in droplet dispersion patterns were observed. The air turbine produced directional spread along the turbine head axis, the US device generated radial dispersion across the operative field, and the implant motor produced substantially less droplet spread than either of the other instruments.

### Evaluation of droplet spread toward the operator

**Fig. 5:**
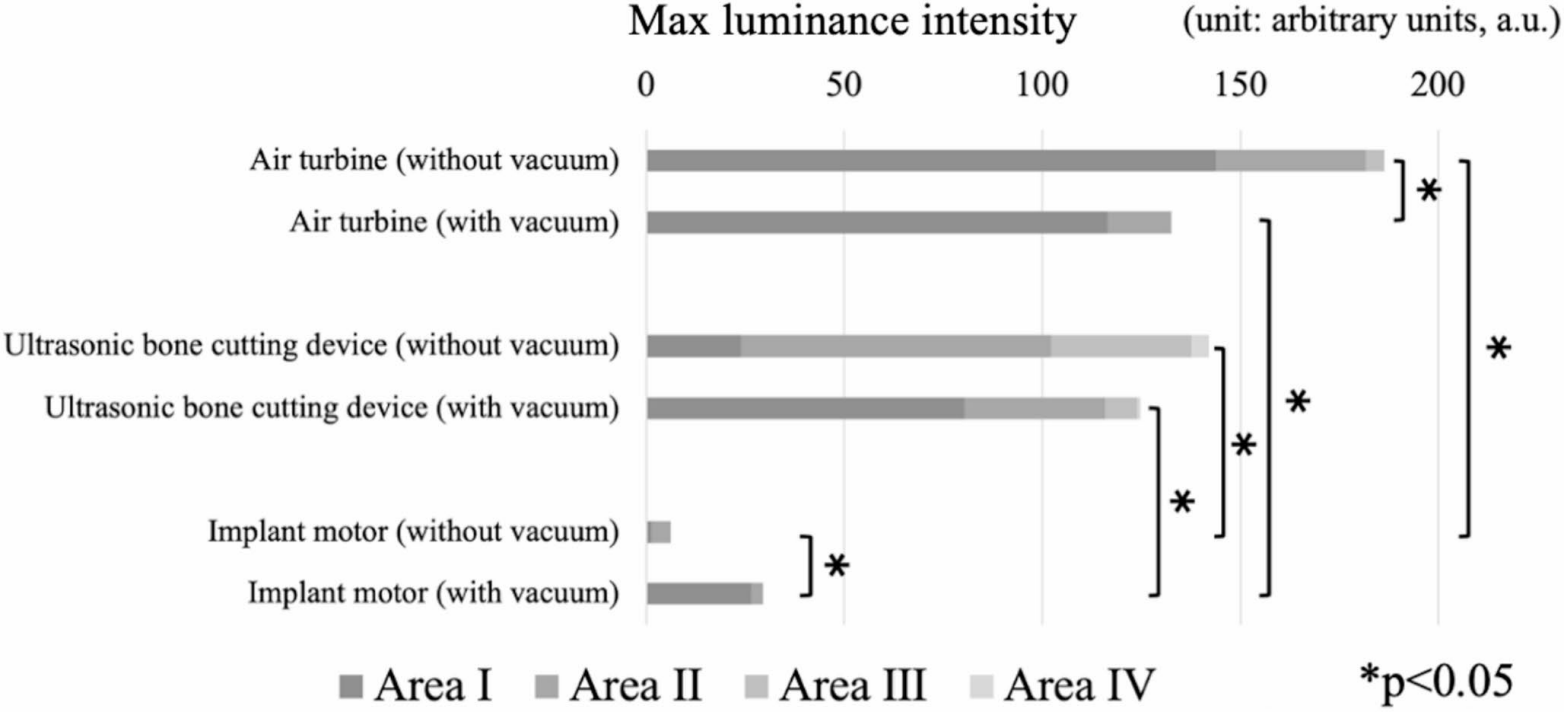
Average maximum luminance intensity for each instrument (*n* = 9) measured by high-speed imaging of laser-illuminated droplets, asterisks indicate *p* < 0.05 versus air turbine handpiece

Using water-sensitive paper and CW laser imaging, droplet dispersion toward the operator’s position was quantified. Maximum luminance intensity analysis and anatomical area classification were applied as described above. Results are summarized in Fig. 5.

The air turbine exhibited the highest luminance intensity, while the implant motor showed significantly lower intensity than both the turbine and US device. Differences in the spatial distribution of maximum luminance were also noted between the turbine and US device (Areas I vs. II).

When the extraoral vacuum was activated, overall luminance decreased for the turbine and US device, whereas a relative increase was observed with the implant motor. Furthermore, activation of the extraoral vacuum shifted the highest luminance intensity closer to Area I.

### CW laser comparison of incisors and molars

**Fig. 6:**
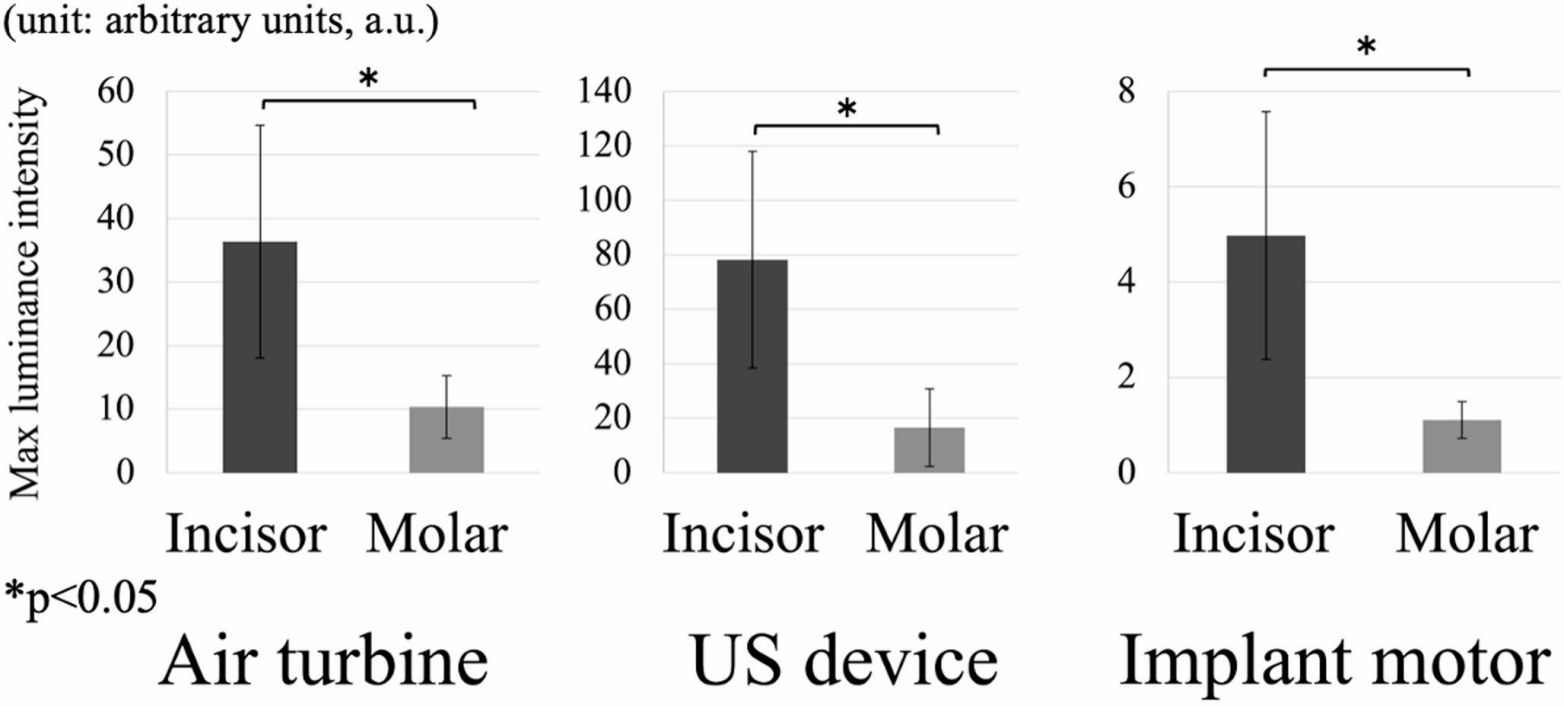
Average maximum luminance intensity at analysis area II (mouth) comparing incisor and molar sites for each instrument (*n* = 9) measured by high-speed imaging of laser-illuminated droplets, asterisks indicate *p* < 0.05 versus incisor site

Figure 6 compares maximum luminance intensity between anterior and molar regions (Area II). Across all instruments, intensity was significantly lower in the molar region (78.5 ± 5.2) compared to the anterior teeth (112.3 ± 6.4; *p* < 0.05, Student’s t-test).

### Water-sensitive paper analysis

1. Extraoral vacuum site.
  When paper was placed near the extraoral vacuum inlet, droplet adhesion increased with use of the air turbine (mean 62 droplets) and US device (mean 49 droplets) when the vacuum was activated (Fig. 7). No droplet adhesion was observed with the implant motor under any condition.

**Fig. 7:**
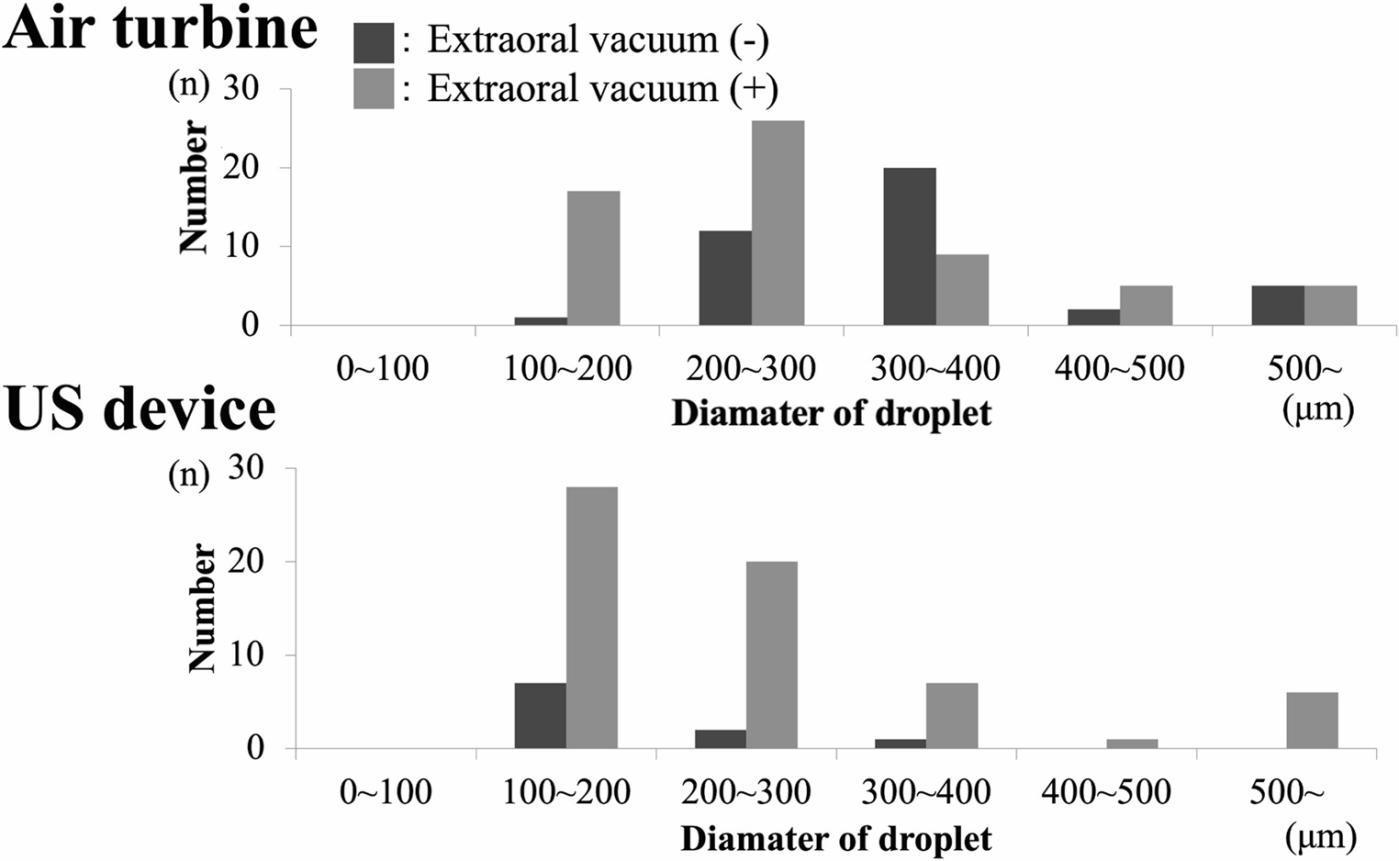
Histogram of droplet size distribution in the extraoral vacuum area, (**A**) air turbine handpiece, (**B**) ultrasonic bone-cutting device, (**C**) implant motor, x-axis droplet diameter (µm), y-axis droplet count, data from water-sensitive paper image analysis (*n* = 3 trials)
2. Patient forehead site.
  At the forehead site, activation of the extraoral vacuum reduced droplet adhesion for both the air turbine (mean 21 droplets) and US device (mean 17 droplets) (Fig. 8). Again, no droplets were detected with the implant motor.

**Fig. 8:**
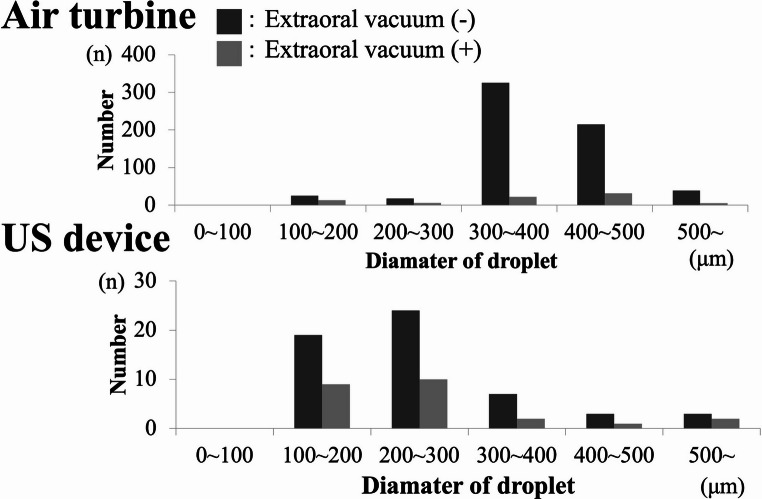
Histogram of droplet sizes on the patient’s forehead, panels (**A**)–(**C**) correspond to the instruments in Fig. 7, droplet diameter and frequency quantified from water-sensitive paper staining
3. Operator mask site.
  At the operator mask site, the number of adhered droplets was consistently lower than at the other two locations (Fig. 9). With the US device, no droplets were detected when the extraoral vacuum was activated. Similarly, no adhesion was observed with the implant motor under any condition.

**Fig. 9:**
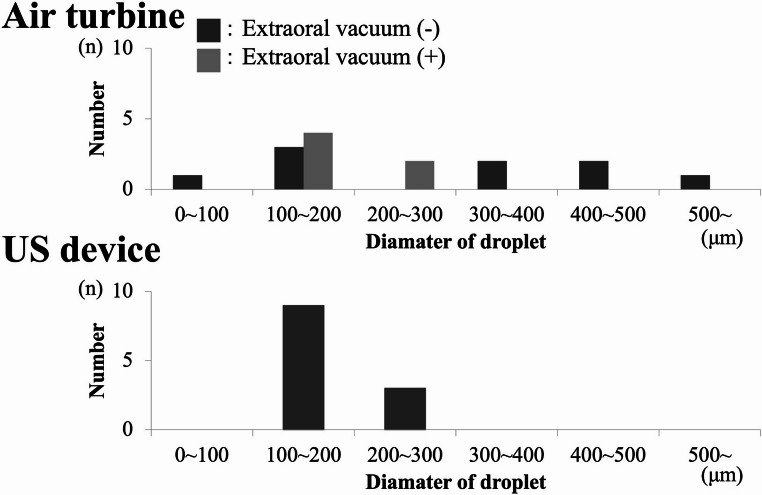
Histogram of droplet sizes on the operator’s mask, panels (**A**)–(**C**) correspond to the instruments in Fig. 7, results show droplet counts by size category from three experiments

## Discussion

While the three instruments studied—air turbine, ultrasonic device, and implant motor—are used for different clinical tasks, comparing them allows evaluation of how their distinct mechanical and irrigation systems affect aerosol and droplet production. The air turbine represents high-speed rotational devices with strong air propulsion, the US device exemplifies vibrational instruments, and the implant motor reflects a low-speed, high-torque system with minimal air discharge. This comparison provides a stratified assessment of infection risk associated with commonly used instruments in clinical dentistry.

The COVID-19 pandemic highlighted the established role of respiratory droplets and direct contact in SARS-CoV-2 transmission, whereas the contribution of aerosols is less definitively understood. Here, aerosolized particles under water irrigation were assessed by luminance intensity, a method that cannot fully distinguish between larger droplets (>20 μm) and finer aerosols (≤ 10 μm) [12, 13]. Nonetheless, the experiments revealed distinct differences in the quantity and distribution of dispersed material among the three instruments, as well as the modifying effect of extraoral vacuum use.

Guidelines such as those from the CDC and ECDC advocate multi-layered infection control measures, emphasizing both high-efficiency suction and careful choice of instruments [14]. Our findings are consistent with existing evidence that high-speed air turbines produce markedly greater aerosol output than lower-speed systems such as implant motors [15]. This distinction has clinical implications: in aerosol-sensitive environments, procedures performed with implant motors may represent a lower infection risk.

Compared with the air turbine and US device, the implant motor generated significantly less dispersion. This is attributable to fundamental design differences. The air turbine generates fine droplets by atomizing coolant with compressed air [16]. In contrast, the implant motor uses a pump-based irrigation system without air atomization, while the ultrasonic device creates secondary atomization due to vibrations at its tip [17]. These differences suggest that implant surgery, typically performed with an implant motor, appears less prone to droplet exposure than general dental procedures.

Anatomical site also influenced droplet behaviour. Treatments performed in the molar region resulted in less diffusion compared with anterior teeth, likely due to partial coverage of the instrument tip by surrounding mucosa. Clinically, this may also inform decisions in autogenous bone harvesting: previous studies have shown that the mandibular ramus not only produces less patient discomfort than the chin region [18], but may also reduce droplet spread when using a US device.

The protective effect of the extraoral vacuum was evident in reducing aerosol and droplet dispersal, consistent with prior reviews of air-cleaning and suction systems [19]. Although water-sensitive paper analysis did not reveal statistically significant reductions, directional decreases in droplet deposition were observed, particularly toward the patient’s head. Nonetheless, practical limitations exist in clinical settings due to positioning and operator accessibility. Other studies emphasize the importance of optimal positioning of the suction port relative to the treatment site [20, 21]. Thus, future work should focus on determining clinically feasible configurations that maximize droplet capture without interfering with procedures.

This study has some limitations. First, droplets and aerosol generated during the simulated dental treatments were expressed as luminance intensity, though it was not possible to determine whether they were directly dispersed into the air from the instruments or through the oral cavity of the patient. Furthermore, only one patient position was used and measurements were obtained with the extraoral vacuum in a single position, thus it will be important to conduct tests with different patient and extraoral vacuum positioning in the future. Finally, for examining the infectivity of droplets, it is necessary to know their vector. More detailed examinations including use of three-dimensional vectors will improve the accuracy of risk assessment during dental treatment.

Moreover, because this study was conducted under simulated experimental conditions rather than in actual clinical settings, the findings may not fully capture the complexity of real patient scenarios, such as variations in oral anatomy, patient movement, and operator technique. Future clinical studies are required to validate and expand upon these experimental results.

## Conclusion

Dental implant surgery using an implant motor generates substantially less droplet diffusion than procedures involving an air turbine or US device. Moreover, use of an extraoral vacuum effectively reduces droplet spread during dental procedures. These findings suggest that implant surgery, particularly when performed with an extraoral vacuum, may present a comparatively lower risk of airborne pathogen transmission in clinical practice.

## Data Availability

No datasets were generated or analysed during the current study.
